# TRH Analog, Taltirelin Improves Motor Function of Hemi-PD Rats Without Inducing Dyskinesia via Sustained Dopamine Stimulating Effect

**DOI:** 10.3389/fncel.2018.00417

**Published:** 2018-11-13

**Authors:** Cong Zheng, Guiqin Chen, Yang Tan, Weiqi Zeng, Qiwei Peng, Ji Wang, Chi Cheng, Xiaoman Yang, Shuke Nie, Yan Xu, Zhentao Zhang, Stella M. Papa, Keqiang Ye, Xuebing Cao

**Affiliations:** ^1^Department of Neurology, Union Hospital, Tongji Medical College, Huazhong University of Science and Technology, Wuhan, China; ^2^Department of Neurology, Renmin Hospital of Wuhan University, Wuhan, China; ^3^Yerkes National Primate Research Center, Emory University School of Medicine, Atlanta, GA, United States; ^4^Department of Neurology, Emory University School of Medicine, Atlanta, GA, United States; ^5^Department of Pathology and Laboratory Medicine, Emory University School of Medicine, Atlanta, GA, United States

**Keywords:** Parkinson’s disease, TRH, Taltirelin, L-DOPA, dopamine, tyrosine hydroxylase

## Abstract

Thyrotropin-releasing hormone (TRH) and its analogs are able to stimulate the release of the endogenic dopamine (DA) in the central nervous system. However, this effect has not been tested in the Parkinson’s disease (PD), which is characterized by the DA deficiency due to the dopaminergic neurons loss in the substantia nigra. Here, we investigated the therapeutic effect of Taltirelin, a long-acting TRH analog on 6-hydroxydopamine-lesioned hemi-Parkinsonian rat model. 1–10 mg/kg Taltirelin i.p. administration significantly improved the locomotor function and halted the electrophysiological abnormities of PD animals without inducing dyskinesia even with high-dose for 7 days treatment. Microdialysis showed that Taltirelin gently and persistently promoted DA release in the cortex and striatum, while L-DOPA induced a sharp rise of DA especially in the cortex. The DA-releasing effect of Taltirelin was alleviated by reserpine, vanoxerine (GBR12909) or AMPT, indicating a mechanism involving vesicular monoamine transporter-2 (VMAT-2), dopamine transporter (DAT) and tyrosine hydroxylase (TH). The *in vivo* and *in vitro* experiments further supported that Taltirelin affected the regulation of TH expression in striatal neurons, which was mediated by p-ERK1/2. Together, this study demonstrated that Taltirelin improved motor function of hemi-PD rats without inducing dyskinesia, thus supporting a further exploration of Taltirelin for PD treatment.

## Introduction

Parkinson’s disease (PD) is a neurodegenerative disease with the second high incidence among elderly over the age of 65. The main “Off” symptoms of PD patients, such as bradykinesia and rigidity, are closely associated with oscillation at the high β band (25–35 Hz) between the motor cortex and the basal ganglia (Jenkinson and Brown, 2011; Li et al., 2012; Dupre et al., 2016), which is usually accompanied by a pathological burst firing pattern of projecting neurons in the motor cortex or striatum (Singh et al., 2016). These abnormal electrophysiological features are the prominent manifestation of disordered basal ganglia neural circuits as a result of the significant loss of dopaminergic neurons in the substantia nigra (SN) and accordingly dopamine (DA) deficiency in the striatum. Thus, the symptoms not only can be halted by deep brain stimulation (DBS) (Li et al., 2012), which directly targets the oscillation, but also can be largely alleviated by pharmacological DA substitution, such as the administration of DA precursor, L-DOPA (Dupre et al., 2016; Kuhn et al., 2017). L-DOPA is a classic medicine used in relieving the symptoms of PD, however, it is gradually recognized that sub-chronic use of L-DOPA is toxic to dopaminergic neurons (Burbulla et al., 2017), and what’s more worrying, L-DOPA-induced dyskinesia (LID) severely damages the life quality of patients and is hard to managed once appears (Calabresi et al., 2010). Thus, novel targets and drugs for the treatment of PD are in urgent need.

Thyrotropin-releasing hormone (TRH) is widely known as a metabolism-regulating endocrine hormone acting through the hypothalamus–pituitary–thyroid axis (HPT axis). However, another role of TRH as a neuropeptide is underestimated (Gary et al., 2003; Khomane et al., 2011). Multiple studies have reported that TRH could promote DA release both *in vitro* (Nishikawa et al., 1993) and *in vivo* (Crespi et al., 1986; Kalivas et al., 1987; Puga et al., 2016). A single injection of TRH increased DA release in striatum by 240% (Kreutz et al., 1990). In addition, a study once shown that subcutaneous injection of TRH sustained-release microparticles significantly improved motor function of encephalitis-induced PD model rats (Ogata et al., 1998). However, TRH has the intrinsic shortcomings such as short half-life, poor lipophilicity and strong HPT axis stimulating effect, which severely restrict its application (Kinoshita et al., 1998). Taltirelin (TA-0910, Ceredist^®^), an oral-effective TRH analog, has 10 to 100 times more potent central nervous system (CNS) stimulant activity and eight times longer duration than TRH. More importantly, Taltirelin has been approved in the treatment of spinocerebellar degeneration (SCD), which makes it highly promising in exploring more applications of TRH family (Kinoshita et al., 1994, 1998). In addition to the anti-ataxic (Nakamura et al., 2005), neuroprotective (Urayama et al., 2002; Veronesi et al., 2007) and analgesic effect (Tanabe et al., 2009), Taltirelin is also able to stimulate DA release (Fukuchi et al., 1998). Is there any benefit or any superiorities over L-DOPA of Taltirelin in treating PD model animals, and what are the possible underlying mechanisms? These issues all await further investigations.

In this study, we established steady *in vivo* electrophysiological recording system of PD model animals to explore and compare the efficacy of Taltirelin and L-DOPA, *in vivo* and *ex vivo*. Our study showed that Taltirelin gently and persistently promoted DA release in the CNS thus alleviated the locomotor disorder and related abnormal electrical activities of PD rats, without inducing dyskinesia even in sub-chronic or high-dose use, thus providing the theoretical basis for a novel PD therapy with Taltirelin.

## Materials and Methods

### Animals

Seven-week-old male Sprague-Dawley (SD) rats (Beijing HFK Bioscience Co., Ltd., China) weighing 230–250 g were used in this experiment. The animals were housed with food and water provided *ad libitum*, 12 h light/dark cycle, constant temperature and humidity. Animal use and care were conformed to the recommendations of the Guidelines of Laboratory Animals Ethics of Tongji Medical College, Huazhong University of Science and Technology. The protocol was approved by the Ethics Committee of Huazhong University of Science and Technology.

### Stereotaxic Surgery

#### 6-OHDA Lesion

Rats were anesthetized with chloral hydrate (7%, 5 ml/kg, i.p.) and secured on the stereotaxic apparatus (RWD Life Science Co., Ltd., China). A temperature controller system was used to maintain body temperature at 37°C. A total dose of 16 μg of 6-hydroxydopamine (6-OHDA, Sigma, United States) dissolved in 4 μl of sterile 0.9% saline and 0.02% ascorbic acid was injected into the right medial forebrain bundle (MFB) via a 10-μl microsyringe at a rate of 0.5 μl/min at the following stereotactic coordinates: anteroposterior (AP), -4.4 mm; mediolateral (ML), -1.5 mm; and dorsoventral (DV), 7.8 mm from dura (Chen et al., 2017). The microsyringe was maintained in place for 5 min before retracting. After 2 weeks of recovery, apomorphine-induced contralateral rotation behavior was assessed.

#### Microelectrode Implantation

After apomorphine induced rotation test, eight near-complete lesioned rats and two intact rats received the implantation surgery. Two multichannel microwire electrode recording arrays, each constructed of eight stainless steel microwires (Stablohm 675, 35 μm in diameter, heavy formvar coated, arranged in 4^∗^2, Bio-Signal Technologies, McKinney, TX, United States) were targeted at the layer V of the primary motor cortex (MI, center coordinates: AP, +2.5 mm; ML, -3 mm; DV, -1.6 mm from dura) (Li et al., 2012) and dorsolateral striatum (DLS, center coordinates: AP, +0.2 mm; ML, -3.8 mm; DV, -3.5 mm from the dura) (Halje et al., 2012) in the lesioned side. Four stainless steel screws were firmly attached to the skull for electrode anchoring, and an additional ground wire was connected to one of them for reference. At the end of the procedure, the whole electrode array was secured with dental cement.

### Behavioral Assessment

#### Apomorphine-Induced Rotation Test

The efficacy of the dopaminergic lesion was tested by measuring contralateral turning behavior with an acute subthreshold dose of apomorphine (0.05 mg/kg s.c.) 2 weeks post surgery. Rats that exhibited more than 200 turns contralateral to the lesion side in 30 min were considered to be compatible with the model of near-complete lesion and were selected for further study (Boldry et al., 1995).

#### Adjusting Step Test

The hindlimbs and contralateral forelimb were slightly lifted up, with only the to-be-examined forelimb touching the table. The rat was moved slowly along the table with speed of 90 cm in 5 s. The number of adjusting steps of each forelimb in the forward directions was counted for three times and took the average (Pinna et al., 2007; Chen et al., 2017).

#### Abnormal Involuntary Movements (AIMs) Score

After injection of L-DOPA (12 mg/kg + benserazide 6 mg/kg, i.p.) or Taltirelin (1 or 5 mg/kg), AIMs were evaluated for 1 min every 20 min for a total 140 min following L-DOPA treatment, using the validated AIMs scale. Axial, limb and orofacial (ALO) dyskinesia were graded, respectively, from score 0 to 4: 0 = absent; 1 = occasional, present during less than half min; 2 = frequent, present during more than half min; 3 = continuous but interrupted by strong sensory distraction; 4 = continuous, not interrupted by strong sensory distraction. All the scores were graded by one experienced researcher during each experiment, while behaviors of animals were also recorded using a video camera for necessary reviews (Lundblad et al., 2004; Chen et al., 2017).

### *In vivo* Electrophysiological Recordings and Analysis

#### Data Recordings

Both the local field potentials (LFPs) and extracellular single-unit activity in MI and DLS were recorded simultaneously using 16-channel Zeus^®^ system (Bio-Signal Technologies, McKinney, TX, United States). Broadband (0.3 Hz–7.5 kHz) neural signals were simultaneously recorded (16 bits @ 30 kHz). Spike and LFP bands were extracted with high-pass (300 Hz) and low-pass (200 Hz) filters, respectively. Real-time spike sorting was performed using principal component analysis (PCA). LFP were down sampled to 1 kHz. Data recorded from intact rats contained 10 min of free moving state. For the hemi-Parkinsonian rats, the recorded data included 10 min of basal line, 120 min continuous data post administration of L-DOPA or at least 10 min data at different time point during 8 h observation period after Taltirelin administration.

#### Spectral Analysis of LFP

Local field potential in MI and DLS was assessed in two frequency ranges: high β (25–35 Hz) and high γ (70–110 Hz) using NeuroExplorer 5.107 software (Nex Technologies, Littleton, MA, United States) as previously described with modification (Li et al., 2012). Briefly, epochs of 120 s, representative of each sample and free of major artifacts, were used to calculate LFP power. The spectrum value was normalized as log of raw power spectral density (PSD) from 0.5 to 200 Hz, which was calculated using Fast Fourier Transform with Hanning window function, shifting each 0.05 s with 50% window overlap. The frequency block was set at 512 at 0.2 Hz resolution.

#### Single-Unit Spike Sorting and Classification

Single-unit spike sorting was performed with Off-Line Spike Sorter 2.8.5 software (Plexon Inc., Dallas, TX, United States) using a combination of automatic and manual sorting techniques. The first three principal components (PCs) of all waveforms recorded from each channel were depicted in 3-dimensional (3D) space. Automatic clustering techniques (K-means clustering and valley seeking methods) were used to produce an initial separation of waveforms into individual cluster. Each cluster was then checked manually to ensure that the cluster boundaries were well separated and spike waveforms were consistent. Pyramidal projection neurons (PNs) and interneurons (INs) in MI (Li et al., 2012), and striatal projection neurons (SPNs), tonically active interneurons (TANs), and fast spiking interneurons (FSIs) in DLS (Singh et al., 2018) were distinguished according as described previously (Supplementary Figure S1).

### Discharge Pattern Analysis

Burst discharge was quantified using Legendy surprise (probability) method with the Poisson surprise threshold at 5 using NeuroExplorer. Parameters for burst detection were based on typical short epochs of spiking that could be unequivocally classified as burst activity. Limits were as follows: maximal interval to start (10 ms), maximal interval to end bursts (10 ms), minimal interburst interval (20 ms), and minimal number of spikes or burst duration (three spikes and 20 ms) (Singh et al., 2016).

### Assessing Neurotransmitters Release by Microdialysis

A self-made concentric circular microdialysis probe (dialysis membrane, RC, cut-off molecular weight: 20 kDa, Union Carbide; membrane length: 3 mm, outside diameter 1.5 mm) (Supplementary Figure S2) was inserted into the lesioned DLS (center coordinates: AP, +0.2 mm; ML, -3.8 mm; DV, -3.5 mm from the dura) under anesthesia with isoflurane (1.5%) (Yan et al., 2016). The sterile artificial cerebrospinal fluid (aCSF) (140 mM NaCl, 3 mM KCl, 1.3 mM, CaCl_2_, 0.9 mM MgCl_2_, 0.27 mM NaH_2_PO4, 1.2 mM Na_2_HPO4, 3.4 mM D-glucose, pH 7.4) were perfused through the microdialysis probe with a constant flow rate of 2.0 μl/ml using a CMA 402 infusion pump (Harvard Apparatus, Holliston, MA, United States). After a stabilization period of 1 h perfusion, two baseline samples of dialysate (0 h) were obtained and then animals were injected i.p. with either saline, 250 mg/kg α-methyl-dl-tyrosine (AMPT, Sigma, United States) (Butcher et al., 1988), 5 mg/kg reserpine (MedChemExpress, United States) (Butcher et al., 1988; Kannari et al., 2000) or 20 mg/kg vanoxerine dihydrochloride (GBR12909; MedChemExpress, United States) (Budygin et al., 2000). Two hours later, 5 mg/kg Taltirelin was injected. Samples were collected at 0.5 h, 2 h, and 3 h of the whole microdialysis experiment. One part of the antioxidative mixture (100 mM acetic acid, 3.3 mM L-cysteine, 0.27 mM Na_2_EDTA, 12.5 μM ascorbic acid, pH 3.2) is added to four parts of dialysate (Van Schoors et al., 2015), which was immediately stored at -20°C until DA and its metabolites measurement by HPLC.

### High Performance Liquid Chromatography Coupled With Electrochemical Detection (HPLC-ECD) Analysis of Neurotransmitters

The level of DA, its metabolites 3,4-dihydroxyphenylacetic acid (DOPAC), or homovanillic acid (HVA) and another monoamine neurotransmitter norepinephrine (NE) of rat tissues or dialysates were analyzed by HPLC-ECD as following: For tissue samples, at different timepoint (0, 0.5, 1, and 2 h) after the i.p. injection of L-DOPA (12 mg/kg, with benserazide 6 mg/kg) or 5 mg/kg Taltirelin. Cortex or striatum of each animal were hand-dissected and tissues of 30–80 mg were homogenized in 300 μl of 0.1 M HClO_4_ (0.1% L-cysteine) and centrifuged at 18,000 × *g* for 15 min at 4°C; supernatants were collected and stored at -20°C. The mixed standards of DA, DOPAC and NE were prepared by diluting each stock solutions (1 mg/ml, in 0.1 M HClO_4_ with 0.1% L-cysteine) with the mobile phase (3 mM sodium heptanesulfonate, 100 mM sodium acetate, 85 mM citric acid, 0.2 mM EDTA, and 8% methanol) into 2.1, 6.2, 18.5, 55.6, 166.7 or 500 ng/ml. For dialysate samples, mixed standards (2.5, 5, and 10 ng/ml) of DA, DOPAC and HVA are prepared by diluting the stock standard solutions (1 mg/ml, aCSF: antioxidative mixture = 4:1) with the mobile phase. Samples and standards (30 μl) were injected into the Waters 510 HPLC system equipped with HPLC column (Zorbax C18, 4.6 mm^∗^25 cm, particle size 5 μm; Agilent, Germany). The mobile phase was delivered at 1 ml/min flow rate; column temperature was 17°C; electrodes potential was 700 mV. Detection of compounds was performed with HP1049i ECD detector. The concentration of each sample was obtained from the standard curve generated by plotting concentration of the standards against respective peak area.

### Serum Thyroid Hormone Measurement

Blood was collected at 2 h post-administration of drugs and placed in 4°C overnight. Then the blood was centrifugated at 3,000 rpm for 15 min, and serum was collected for further analysis. The levels of thyroid stimulating hormone (TSH), free and total thyroxine (T4) or triiodothyronine (T3) were measured using ELISA kit, respectively (ALPCO) according to the manufacturer’s protocol. Briefly, 50 μL of serum samples or reference was pipetted into the assigned wells, followed by T3/T4 enzyme-conjugated solution with 1 h of incubation. The mixture was removed, and the plate was washed several times with water; then, working substrate solution was added to each well for reaction, and after 20 min in the dark, the reaction was stopped by adding 3 M HCl. Absorbance was read at 450 nm. The corresponding concentrations were calculated based on the standard curve.

### Primary Neonatal Rat Striatal and Cortex Neurons Culture

Primary rat striatal and cortex cultures were prepared as described previously (Beaudoin et al., 2012). In brief, the striatum and cortex was dissected from P1-3 mouse pups. The isolated tissues were then chemically and mechanically dissociated into single cell suspensions. Cells were plated into 6-well plates coated with 0.5 mg/ml poly-L-lysine at a density of 5.5–6 × 10^5^/well. Incubate the cells with plating medium: DMEM/F-12 medium (Hyclone, United States) with 20% FBS (Gibco, United States) and 100 units/ml of penicillin and streptomycin for 4 h and replace the medium with maintenance medium: Neurobasal medium (Gibco, United States) with 2% B-27 supplement (Gibco, United States), 2 mM glutamine (Gibco, United States) and 100 units/ml of penicillin and streptomycin. Cultures were maintained in a humidified incubator at 37°C/5% CO_2_ for 3 days *in vitro* prior to further studies.

### Western Blot Analysis

For animal brain tissues, striatum was dissected and homogenized with micro-tissue-grinders (Tiangen, OSE-Y30) in ice-cold enhanced RIPA lysis buffer [50 mM Tris, pH 7.4, 150 mM NaCl, 1% Triton X-100, 1% sodium deoxycholate, 0.1% SDS, 5 mM EDTA, 2 mM Na3VO4, 1 mM PMSF, 10 mM NaF, and a cocktail of protease inhibitors (Roche, United States)]. For cellular culture, cells were scraped down by a cell scraper and lysed in lysis buffer. Brain or cellular lysates were centrifuged at 12,000 × *g* at 4°C for 15 min and the protein concentrations were determined by a BCA assay kit (Pierce, Rockford). The supernatants were boiled for 10 min with 1% SDS loading buffer. After SDS-PAGE, the samples are transferred to a PVDF membrane (Millipore, United States), blocked with 5% non-fat milk for 1 h at room temperature, and incubated overnight at 4°C with primary antibodies against following proteins: tyrosine hydroxylase (TH) (1:1000, Proteintech, 25859-1-AP), p44/42 MAPK (ERK1/2) (1:2000, Cell Signaling, #4695), phospho-p44/42 MAPK (ERK1/2) (1:2000, Cell Signaling, #4370), ΔFosB (1:500, Cell Signaling, #9890), GAPDH (1:2000, AntGene, ANT012). The membranes were washed with TBST, then incubated with HRP-conjugated anti-rabbit secondary antibody (1:3000, AntGene, ANT020) for 1 h at room temperature. After washing, the membrane was visualized with an ECL detection kit (Thermo Scientific) in the Bio-Rad imaging system. Bands intensities were analyzed with ImageJ software.

### Immunohistochemical and Immunofluorescent Staining

#### Immunohistochemical Staining (IHC)

Animals were deeply anesthetized and transcardially perfused with 0.9% saline, followed by 4% paraformaldehyde (PFA) in 0.1 M PBS (pH 7.4). Brain were dissected and fixed in 4% PFA overnight at 4°C. After fixation, tissues were transferred to 70% ethanol and processed for paraffin sectioning (4 μm/section). Sections were mounted on glass slides, deparaffinized by xylene, dehydrated in graded ethanol solutions, baked in the basic antigen retrieval buffer (pH = 6.0), and washed with phosphate buffer (pH 7.4). After washing, sections were blocked with 3% bovine serum albumin (BSA) and then incubated with diluted primary antibody in a humidified chamber overnight at 4°C. The following primary antibodies were used: anti-FosB antibody (1:100, Santa Cruz, sc-48, detecting FosB-ΔFosB), anti-TH antibody (1:750, Abcam, ab112). Sections were washed and subsequently incubated with biotinylated goat anti-rabbit IgG, then HRP labeled streptavidin fluid, followed by DAB solutions, counterstained with Harris hematoxylin, dehydrated in graded ethanol solutions, and eventually cover slipped. Images were collected through an Olympus camera connected to the microscope at the same light intensity, and analyzed using Image-Pro Plus software.

#### Immunofluorescent Staining (IF)

For brain slices, immunofluorescent staining shared a same procedure with immunohistochemistry staining before secondary antibodies incubation. For cellular cultures, sterile coverslips were placed in 24-well plates and coated with PLL before cells were plated on the coverslips. After intervention, cells were first washed with PBS and fixed with 4% PFA. Then cells were permeablized with 0.2% Triton X-100 and blocked with 5% BSA. Incubate brain slices or cell slides with following primary antibodies overnight at 4°C: anti-DOPA decarboxylase (AADC) antibody (1:250, Abcam, ab3905), anti-dopamine transporter (DAT) antibody (1:50, Santa Cruz, sc-32258), anti-TH antibody (1:750, Abcam, ab129991), anti-MAP2 antibody (1:500, Servicebio, Wuhan), anti-GABA antibody (1:200, Abcam, ab8891), anti-dynorphin antibody (1:50, Santa Cruz, sc-46313), anti-enkephalin (1:100, Abcam, ab150346). After being washed, sections were incubated in dark with an appropriately diluted Alexa 488- or Cyanine 3-coupled secondary antibodies followed by DAPI staining nucleus for 10 min. Coverslips were mounted on slides with one drop per coverslip of antifade mounting medium (Beyotime, China). Images were collected using fluorescence microscopy with image manipulation software, and analyzed using Image-Pro Plus software.

### Statistic Analysis

Data were analyzed using one-way analysis of variance (ANOVA) followed by Tukey HSD or LSD *post hoc* tests for multiple comparisons between groups, and Student’s *t*-test for comparing two groups. All statistical analyses were performed in SPSS 21.0 software. The significance level was set at *p* < 0.05. Data are presented as mean ± SEM for all results.

## Results

### Taltirelin Improved Motor Function and Normalized Aberrant Electrical Activity of 6-OHDA-Lesioned Rats

**FIGURE 1:**
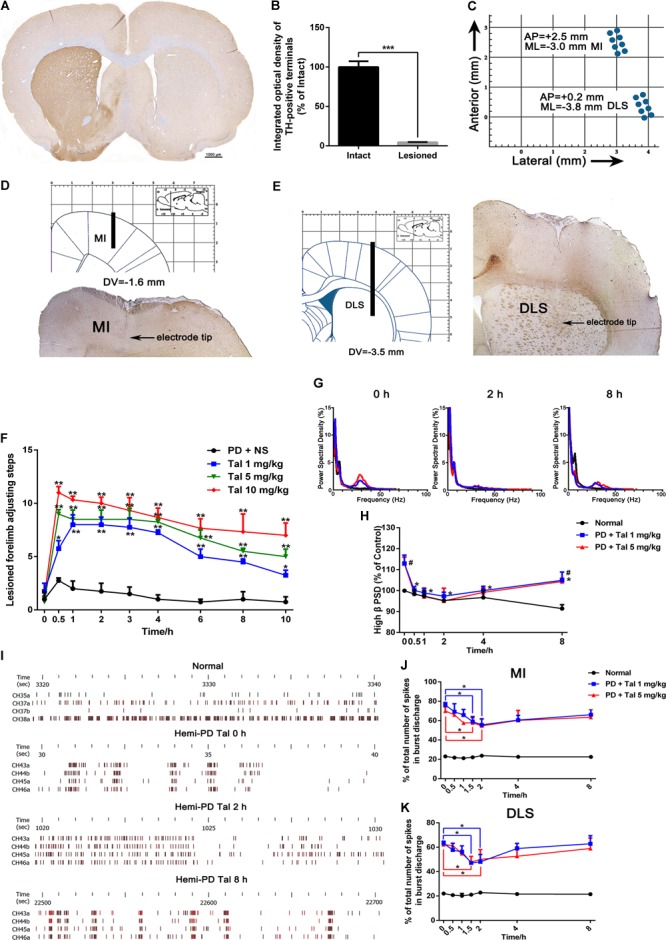
Taltirelin improved motor function and normalized aberrant electrical activity of 6-OHDA-lesioned rats. **(A)** Tyrosine hydroxylase (TH) immunostaining in the striatum of 6-OHDA lesioned rat model. **(B)** Integrated optical density of TH-positive terminals in the intact and lesioned side. *N* = 3. **(C)** Schematics illustrating horizontal positioning of recording electrodes targeting primary motor cortex (MI) or dorsolateral striatum (DLS) relative to bregma. **(D,E)** Coronal plane indicating schematic vertical positions for MI and DLS together with AP positions (left). Typical brain slice showed actual electrode position (right). Electrodes were implanted in the right (lesioned) side. **(F)** Adjusting step tests of PD rats treated with saline or Taltirelin (Tal 1, 5 or 10 mg/kg, i.p.). *N* = 6. **(G)** Typical example of power spectral distribution of local field potential (LFP) after treatment of Taltirelin (1 or 5 mg/kg, i.p.). **(H)** Power of high β oscillation changed with time (a total of 128 LFP data from two intact rats and six hemi-Parkinsonian rats). **(I)** Typical examples of raster plots showing the neuronal discharge patterns of normal or 6-OHDA-lesioned rats. **(J,K)** The % of total number of spikes in burst discharge of PNs or striatal projection neurons (SPNs) (a total of 75 PNs and 78 SPNs from two intact rats and eight hemi-parkinsonian rats). ^#^*p* < 0.05 vs. control; ^∗^*p* < 0.05; ^∗∗^*p* < 0.01; ^∗∗∗^*p* < 0.001. Error bars denote SEM.

The *in vivo* multi-channel electrophysiological recording technology, of good time and spatial resolution, allows real-time recording and analysis of electrophysiological data and animal behavior simultaneously, which makes it a unique tool in studying mechanisms of motor diseases. After the apomorphine-induced rotation tests, complete-depleted (TH-positive dopaminergic terminals in striatum, Figures 1A,B, or dopaminergic neurons in the SN, Supplementary Figure S3, were reduced by more than 90%, *p* < 0.001) animals were implanted with electrodes targeting MI and DLS (Figures 1C–E). When spikes and LFP could be steadily recorded in most of the channels, behavior assessments were performed. In the adjusting step test, the number of steps of the lesioned forelimb of the PD rats significantly decreased compared with the intact forelimb (1.39 ± 0.23 vs. 9.97 ± 0.28, *p* < 0.001), indicating an impaired locomotor function. After Taltirelin 1–10 mg/kg i.p. injection, the bradykinesia of lesioned forelimb was relieved at as early as 0.5 h after injection (5.75 ± 1.5 of 1 mg/kg, 9.50 ± 2.38 of 5 mg/kg, 11.00 ± 1.00 of 10 mg/kg vs. 2.75 ± 0.50 of control, *p* < 0.01). The improving effect maintained for at least 10 h and was dose-dependent (Figure 1F).

Next, the LFP PSD analysis showed that free-moving PD rats exhibited high β band oscillation (25–35 Hz) in the MI and DLS ipsilateral to the lesion, which is a characteristic abnormal electrical activity associated with bradykinesia. Administration of 1 mg/kg or 5 mg/kg Taltirelin caused a significant reduction of the power of the β band oscillation, which approached the normal level 2 h later (86.15 ± 3.94%, 84.27 ± 3.15%, respectively, compared with their baseline, Figures 1G,H). Furthermore, a total of 75 discharge units in the MI and 78 discharge units in the DLS were recorded from 10 rats in this experiment. 32 PNs (44%) and 36 SPNs (46%) were classified for following single unit firing pattern analysis. Consistent with previous studies, there was an increase of burst firing rather a random firing mode of neurons at the lesioned side of the PD rat. Similarly, Taltirelin (1 or 5 mg/kg) significantly reduced the total number of spikes in burst discharge of PNs by 17.06% and 12.32%, or of SPNs by 14.29% and 16.03%, respectively (Figures 1I–K, *p* < 0.05). Therefore, the alleviation of abnormal electrophysiological indicators further supported the finding that Taltirelin relieved the bradykinesia of 6-OHDA-PD rats.

### Acute or Sub-Chronic Administration of Taltirelin Relieved Bradykinesia Without Inducing Dyskinesia

**FIGURE 2:**
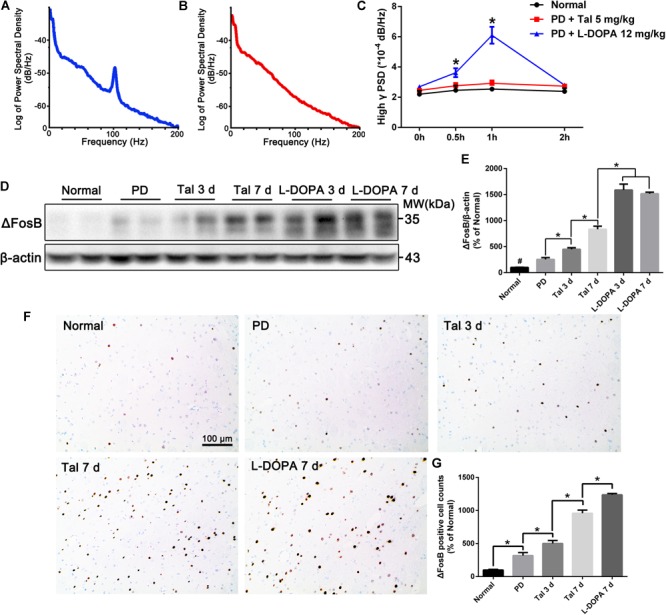
Acute or sub-chronic administration of Taltirelin relieved bradykinesia without inducing dyskinesia. **(A,B)** Power spectral distribution of local field potential (LFP) after treatment of L-DOPA (12 mg/kg, with benserazide 6 mg/kg) or Taltirelin (5 mg/kg, i.p.). **(C)** Power of high γ oscillation changed with time (32 LFP data in MI and DLS of two intact rats and 96 LFP records in six lesioned rats). **(D,E)** Western blot analysis of ΔFosB in the DA-denervated striatum of each group (normal, PD, Tal 5 mg/kg or L-DOPA 12 mg/kg treated for 3 or 7 days). *N* = 3. **(F)** Immunostaining of ΔFosB of lesioned striatum. **(G)** Counts of ΔFosB-positive cells in each group. *N* = 3. ^#^*p* < 0.01 vs. intact or other groups; ^∗^*p* < 0.05; ^∗∗^ *p* < 0.01. *N* = 3. Error bars represent SEM.

L-DOPA-induced dyskinesia is one of the main drawbacks of long-term L-DOPA therapy, which are characterized by involuntary, repetitive orofacial, limb and axial movements. Interestingly, no dyskinesia-related behaviors were observed in the animals treated with Taltirelin even at high, toxic dose (10 mg/kg). In order to confirm the difference between Taltirelin and L-DOPA, we chose a relative high dose (5 mg/kg) of Taltirelin and a high dose (12 mg/kg, with benserazide 6 mg/kg) of L-DOPA which usually induces dyskinesia at first dose. As expected, animals injected i.p. with L-DOPA exhibited obvious dyskinesia as early as 20 min post-administration. Meanwhile, the power of β oscillation decreased with the release of bradykinesia, while the dyskinesia-associated high γ band oscillation (70–110 Hz) arose and intensified as dyskinesia aggravated (increased by 126% compared with baseline at 1 h, *p* < 0.05). On the contrary, the animals injected with Taltirelin did not show any abnormal behaviors or high γ oscillation (Figures 2A–C and Supplementary Figures S4A,B).

ΔFosB is a classic marker of LID, whose level in the DA-deprived striatum is positively related to the severity of LID (Cao et al., 2010) (Supplementary Figures S4C,D). The animals treated with Taltirelin for 7 days showed no signs of dyskinesia, however, there was a moderate but significant elevation of ΔFosB in the lesion striatum (834.40 ± 58.46% of the normal) compared with the short-term (3 days) Taltirelin (449.70 ± 29.15%) or PD control (254.7 ± 34.46%). However, the ΔFosB levels in Taltirelin groups were still lower than the levels in L-DOPA (12 mg/kg) group of 3 days (1588.00 ± 84.70%) or of 7 days (1515.00 ± 30.94%) (Figures 2D,E, *p* < 0.05). The counts of ΔFosB-positive cells in the striatum confirmed the observation in Western blot (PD < Tal 3 days < Tal 7 days < L-DOPA, 318.18 ± 40.45%, 511 ± 32.28%, 957.58 ± 48.48%, 1236.36 ± 21.00%, respectively) (Figures 2F,G, *p* < 0.05).

**FIGURE 3:**
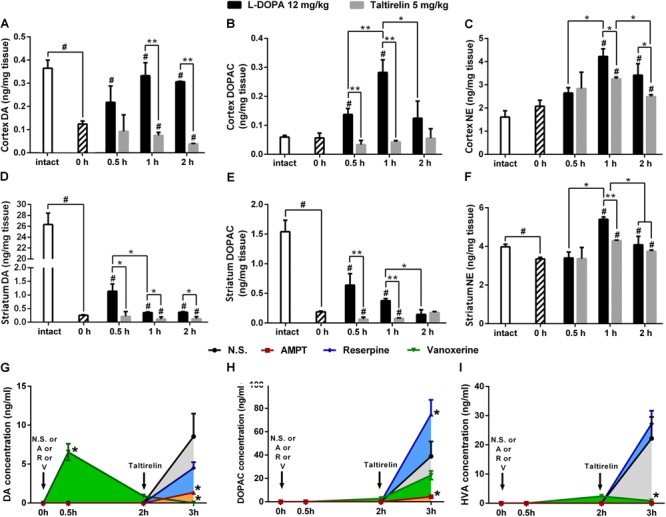
Taltirelin elevated dopamine level in a DA-releasing dependent manner. **(A–F)** The high performance liquid chromatography coupled with electrochemical detection (HPLC-ECD) assessed the intracellular dopamine (DA), 3,4-dihydroxyphenylacetic acid (DOPAC) and norepinephrine (NE) in the cortex and striatum of 6-OHDA lesioned animals treated with L-DOPA or Taltirelin. ^#^*p* < 0.05 vs. 0 h, ^∗^*p* < 0.05 vs. indicated group, ^∗∗^*p* < 0.05 vs. indicated group. **(G–I)** Effects of AMPT (250 mg/kg, i.p.), reserpine (5 mg/kg, i.p.) or vanoxerine dihydrochloride (20 mg/kg, i.p.) on Taltirelin-induced alterations of DA, its metabolites DOPAC and HVA in dialysates collected from lesioned DLS. Taltirelin (5 mg/kg) was injected 2 h after administration of three medications. ^∗^*p* < 0.05 vs. other groups at the same time point. *N* = 3. Error bars represent SEM.

We further assessed the levels of DA, its metabolite, DOPAC and another monoamine neurotransmitter NE in the cortex and striatum of PD rats (Figures 3A–F). As expected, the basal level of DA in the lesioned cortex (0.13 ± 0.02 ng/mg vs. 0.37 ± 0.06 ng/mg, *p* < 0.01) and striatum (0.25 ± 0.03 ng/mg vs. 26.31 ± 2.96 ng/mg, *p* < 0.01) was significantly lower than the intact side. Similarly, DOPAC (0.19 ± 0.02 ng/mg vs. 1.54 ± 0.34 ng/mg, *p* < 0.05) and NE (3.35 ± 0.15 ng/mg vs. 3.98 ± 0.23 ng/mg, *p* < 0.05) also decreased significantly in the striatum. After injection of L-DOPA, DA, DOPAC and NE in both the cortex and striatum increased significantly (*p* < 0.05). However, the concentrations of DA in the lesioned striatum peaked at 0.5 h (1.14 ± 0.46 ng/mg) post-injection, which then decreased with time, while the DA in the cortex peaked at 1 h (0.33 ± 0.10 ng/mg), which was in a better correlation with the LID behaviors and CNS abnormal electrical activities (Figure 2C). On the contrary, Taltirelin induced a slight reduction of DA and elevation of DOPAC in the cortex and striatum, while the trend of NE level is similar to the L-DOPA group though at a lower level. The results in the Taltirelin group seemed contradictory but was consistent with previous studies (Puga et al., 2016). Since the tissue homogenates were mainly composed of intracellular fluids, a decrease in content may suggest the release of these neurotransmitters into the extracellular spaces. To confirm this, we then examined the DA-releasing effect of Taltirelin through microdialysis.

### Taltirelin Elevated Dopamine Level in a DA-Releasing Dependent Manner

Here, we used microdialysis and three DA-releasing-related drugs to confirm the DA-releasing function of Taltirelin and investigate the possible underlying mechanisms. TH inhibitor, AMPT and vesicular monoamine transporter-2 (VMAT-2) inhibitor, reserpine both causes DA depletion and results in very low level of both intracellular and extracellular DA in the striatum. Vanoxerine (GBR12909) is a DA reuptake inhibitor, which elevates DA in striatum after administration but would also deplete DA pool. Results showed that the basal extracellular levels of DA, DOPAC and HVA in the lesioned striatum of rats were too low to detect. As expected, vanoxerine (20 mg/kg, i.p.) caused a sharp increase (6.55 ± 1.07 ng/ml, *p* < 001) and then fall of DA, while AMPT (250 mg/kg, i.p.) and reserpine (5 mg/kg, i.p.) produced no undetectable alteration of DA. As the level of DA stabilized 2 h later, Taltirelin (5 mg/kg, i.p.) was administrated, and DA was elevated to different levels in each groups: N.S. > reserpine > AMPT > vanoxerine (8.54 ± 2.94 ng/ml, 4.51 ± 0.73 ng/ml, 1.36 ± 0.22 ng/ml, 0 ng/ml) (Figure 3G). Thus, the results supported the fact that Taltirelin promotes DA release in DA-depleted striatum. In addition, the lower or even absent DA in groups with pre-treatment further suggested that intracellular DA pool made up a large part of DA released by Taltirelin. As for the metabolites of DA, DOPAC: reserpine > N.S. > vanoxerine > AMPT (75.06 ± 12.22 ng/ml, 38.97 ± 12.67 ng/ml, 22.75 ± 3.70 ng/ml, 4.35 ± 1.09 ng/ml) (Figure 3H); HVA: reserpine > N.S. > vanoxerine > AMPT (27.25 ± 12.22 ng/ml, 22.22 ± 7.34 ng/ml, 0.83 ± 0.13 ng/ml, 0.06 ± 0.01 ng/ml) (Figure 3I). Since inhibition of VMAT-2 by reserpine led to more chances for DA to be exposed and metabolized in the cytoplasm, more DA metabolites were generated accordingly. Interestingly, incomplete blockade by the above DA-releasing inhibitors and less DA in group treated with TH-inhibitors AMPT suggested the existence of a newly synthesized DA pool induced by Taltirelin, which is more meaningful as the number of residual dopaminergic neurons, i.e., the storage of DA, diminishes as the disease progresses, and new source of DA would be very in need. Thus, we next examined the possible ability of Taltirelin to manipulate DA synthesis.

### Taltirelin Elevated the Level of TH Both *in vivo* and *in vitro* in a p-ERK/1/2-Dependent Manner

**FIGURE 4:**
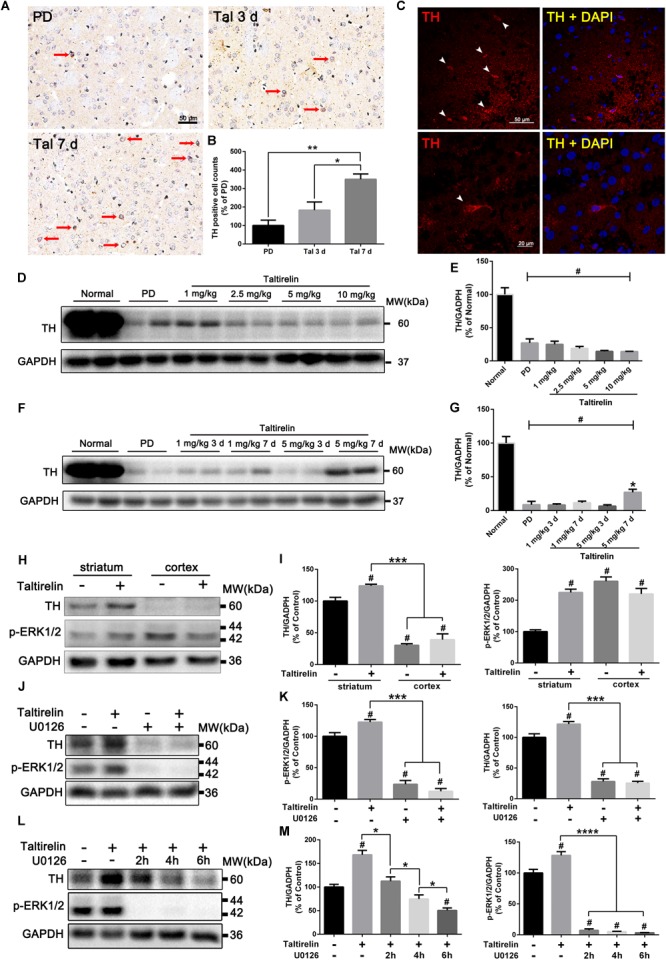
Taltirelin elevated the level of TH both *in vivo* and *in vitro* in a p-ERK/1/2-dependent manner. **(A)** Immunostaining of tyrosine hydroxylase (TH) in the lesioned striatum of each group (PD, Tal 5 mg/kg for 3 days or 7 days). Red arrows indicated TH-positive cells. **(B)** Counts of TH-positive cells. **(C)** Immunofluorescent staining of TH in the lesioned striatum of animals treated with Tal 5 mg/kg for 7 days. White arrows indicated TH-positive cells (top). Higher-magnification photomicrographs of a TH-positive cell (down). **(D,E)** Western blot analysis of TH in the lesioned striatum of each group (normal, PD, Tal 1, 2.5, 5, or 10 mg/kg). **(F,G)** Western blot analysis of TH in the lesioned striatum of each group (normal, PD, Tal 1 or 5 mg/kg continuous administrated for 3 or 7 days). **(H,I)** Western blot analysis of intracellular TH and p-ERK1/2 of the primary rat striatal or cortical neurons treated with Tal 1, 5, or 10 μM. **(J,K)** Western blot analysis of intracellular TH and p-ERK1/2 of the primary rat striatal neurons pre-treated with MEK inhibitor U0126 (10 μM) for 2 h, then incubated with Tal (5 μM) for 24 h. **(L,M)** Western blot analysis of intracellular TH and p-ERK1/2 of the primary rat striatal neurons incubated with Tal (5 μM) for 24 h, then treated with U0126 (10 μM) for 2 h, 4 h, or 6 h. ^#^*p* < 0.01 vs. normal or control; ^∗^*p* < 0.05; ^∗∗^*p* < 0.01; ^∗∗∗^*p* < 0.001; ^∗∗∗∗^*p* < 0.0001. *N* = 3. Error bars represent SEM.

It was once reported that TRH could elevate the activity of TH (Yokoo et al., 1983), but whether the expression of TH could be manipulated by TRH was not reported. We thus stained the DA-deprived striatum slices with TH and found significantly increased TH-positive neurons in animals injected with Taltirelin (5 mg/kg) for continuous 7 days but not in animals with 3-day injection (350.00 ± 28.87%, 183.33 ± 44.10%, respectively, Figures 4A–C; *p* < 0.05). We then quantified the level of TH in animals injected with different doses of Taltirelin for 3 days, but there was no obvious elevation of TH even at high dose such as 10 mg/kg (Figures 4D,E). In contrast, the TH level in the animals injected with Taltirelin (5 mg/kg) for 7 days (27.36 ± 4.58%) was significantly higher than other groups, including Taltirelin (1 mg/kg, 7 days) (7.12 ± 4.43%) (Figures 4F,G; *p* < 0.05). The above results showed that sub-chronic, high-dose Taltirelin could elevate TH level in the lesioned striatum of PD rats. Besides, we also conducted double-staining of TH with other dopaminergic neurons markers, such as AADC and DAT, but found no double-positive neurons (data not shown), indicating that those Taltirelin-induced TH-positive neurons were non-dopaminergic neurons.

We further verified our results *in vitro* using primary neurons. It was reported that there was a 4-fold increase in the activity of TH in the brain during rat embryonic development, while only a 2.5-fold increase after birth (Coyle and Axelrod, 1972). To ensure the consistency, we cultured the neonatal rats of 1–3 days (P1–3) primary striatal neurons. We found that primary striatal neurons cultured for 3 days had basal expression of TH, while the primary cortical neurons had a much lower level of TH. Studies have shown that *ex vivo* cultured itself could induce TH expression (Du and Iacovitti, 1997), which may act as compensation to the loss of DA. On the third day *in vitro*, the primary neurons were treated with Taltirelin (5 μM) for 24 h, and we found TH increased in the primary striatal neurons (136.20 ± 3.47%, *p* < 0.05) but not in the primary cortical neurons. In addition, p-ERK1/2 increased by 124.84% in the primary striatal neurons, but decreased by 15.52% in the primary cortical neurons (Figures 4H,I), indicating that the effect of Taltirelin on TH expression was neuronal species-specific.

The elevation of p-ERK1/2 along with the TH suggested that this signaling molecule may be involved in this process. Therefore, we pre-treated primary striatal neurons with U0126 (10 μM) for 2 h to inhibit MEK, the upstream kinase of ERK1/2, then incubated neurons with Taltirelin (5 μM) for 24 h. As expected, U0126 significantly decreased p-ERK1/2 by 76.28% and TH by 71.93% (Figures 4J,K; *p* < 0.001), indicating that Taltirelin-induced TH expression is p-ERK1/2-dependent. Next, after 24 h incubation of Taltirelin, we treated neurons with U0126 for further 2 h, 4 h, or 6 h. We found that p-ERK1/2 was almost completely blocked after treatment with U0126 for 2 h (7.28 ± 2.36% of control), accompanied by TH decreasing with time (2 h: 112.60 ± 8.97%, 4 h: 74.71 ± 8.67%, 6 h: 50.48 ± 5.29%) (Figures 4L,M; *p* < 0.05), indicating that down-regulating p-ERK1/2 interfered with the maintenance of the level of cellular TH.

**FIGURE 5:**
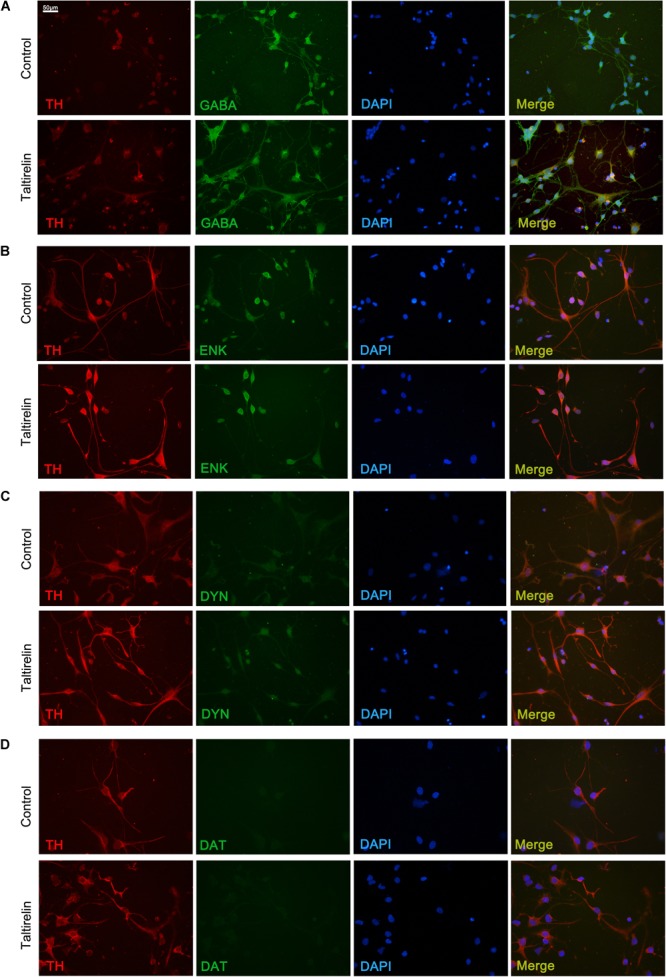
Taltirelin elevated the level of TH in enkephalin-positive neurons *in vitro*. Double immunolabeling images showed co-localization of tyrosine hydroxylase (TH) with GABA **(A)**, enkephalin **(B)**, dynorphin **(C)** or dopamine transporter (DAT) **(D)** in control or Taltirelin treated rat primary striatal neurons. *N* = 3.

In order to determine the species of neurons expressing TH *in vitro*, we co-stained the primary striatal neurons with TH and other markers, such as GABA, enkephalin, dynorphin or DAT. The results showed that the neurons treated with Taltirelin exhibited wider distribution and higher fluorescent intensity of TH, which is consistent with the above results. The SPNs are GABAergic neurons, and we found TH mainly co-located with GABA in neurons treated with or without Taltirelin, indicating that these TH-positive neurons are GABAergic (Figure 5A). GABAergic neurons are divided into two main species: the enkephalin-positive and the dynorphin-positive, each plays different role in basal ganglia neural circuits. In our experiments, TH-enkephalin, but not TH-dynorphin double-positive neurons were observed, suggesting that TH was mainly expressed by enkephalin-positive GABAergic neurons (Figures 5B,C). DAT is another essential enzyme in the synthesis process of DA, but DAT-positive neurons were not found in this experiment (Figure 5D), which was consistent with the results *in vivo* that those new-emerging TH-positive cells were not dopaminergic neurons.

## Discussion

Thyrotropin-releasing hormone and its analogs have long been recognized as DA-releasing agents in CNS (Crespi et al., 1986; Fukuchi et al., 1998; Puga et al., 2016), however, their therapeutic potential toward PD, which is mainly characterized by insufficient DA in striatum, is rarely recognized or discussed thoroughly. Our results showed that Taltirelin (1–10 mg/kg) significantly improved the locomotor function of 6-OHDA-induced hemi-Parkinsonian rat model, alleviated the intensity of bradykinesia-associated high β oscillation and reversed the bursting firing form of projecting neurons in MI and DLS. The beneficial effect maintained for as long as 8 h, which was in consistency with the long half-life of Taltirelin (Kinoshita et al., 1998). Besides, animals exhibited involuntary shaking and repeated jaws action after the injection of Taltirelin above 5 mg/kg (Supplementary video), which may be related to the stimulation of 5-HT system (Miwa et al., 1995) by Taltirelin during high dose use. We also examined and found that Taltirelin (>5 mg/kg) elevated serum level of TT3, TT4, FT3, and FT4 in PD rats (Supplementary Figure S5). Compared with TRH, Taltirelin has much weaker peripheral stimulatory effect (Fukuchi et al., 1998), and it was once reported that prolonged oral administration of Taltirelin of 2.5–20 mg did not significantly alter the pituitary–thyroid axis in subjects with brain stroke (Miyashita et al., 1993). However, it has been reported that hyperthyroidism and hypothyroidism are more common in PD patients than in the general population (Daimon et al., 2013). Thus, the thyroid function of patients should be thoroughly evaluated and adjusted accordingly before administration of Taltirelin if possible in the future.

Impressively, Taltirelin improved locomotor function without inducing dyskinesia-like behaviors for sub-chronic use, which was confirmed by the absence of LID-related high γ band oscillation (70–110 Hz) during the PD symptom-remitting period. Measurement of DA in the brain tissues and dialysates of PD rats by HPLC-ECD confirmed that Taltirelin promoted DA release in a mild and persistent manner. In contrast, after L-DOPA injection, DA sharply elevated in the cortex, and peaked at 1 h then maintained at high concentration, which was in good correlation with the severity of dyskinesia behaviors. Studies once showed that application of dopamine type 1 receptor (D1R) antagonists on the surface of the DA-depleted cortex immediately halted LID symptoms and terminated high γ band oscillation in PD rats (Halje et al., 2012). It is suggested that discontinuous drug delivery due to the short half-life of L-DOPA and the variability in its gastrointestinal absorption are responsible for the non-physiological pulsatile cortical dopamine receptor stimulation and the dopamine receptor hypersensitivity (Halje et al., 2012; Poewe and Antonini, 2015; Antonini et al., 2016). DA agonists, monoamine oxidase (MAO) and catechol-*O*-methyltransferase (COMT) inhibitors, and sustained-effective L-DOPA preparations under development are all aimed at achieving the goal of continuous dopamine receptor stimulation (Poewe et al., 2017). Thus, the long half-life and absence of dyskinesias in sub-chronic and high dose application of Taltirelin do bring benefit to PD treatments. However, the anti-parkinsonian effect of Taltirelin in longer period of time should be investigated in the future, for 7-day sub-chronic scheme is not enough to completely address the long-term impact of Taltirelin.

The mechanism by which TRH promotes the elevation of DA is still unclear so far (Yokoo et al., 1983; Prasad, 1991; Fukuchi et al., 1998). Reserpine irreversibly blocks the uptake of catecholamines (NE, DA and 5-HT) into synaptic vesicles by inhibiting the VMAT-2, and neurotransmitter in the cytoplasm are easily degraded by MAO, thus depleting the vesicular storage of catecholamines (Kannari et al., 2000). Pretreatment with reserpine produced 47.19% reduction in Taltirelin-derived extracellular DA levels, indicating that a large part of the Taltirelin-derived DA release is of vesicular origin. Vanoxerine (GBR12909) usually caused a slow and sustained elevation of extracellular DA by inhibiting DA uptake (Nakachi et al., 1995; Gagnaire and Micillino, 2006), however, in our study, DA increased by vanoxerine returned to their baseline 2 h after administration, which may be due to the deficiency of DA caused by 6-OHDA lesion. The interesting finding here was that vanoxerine almost totally blocked the ability of Taltirelin to increase extracellular DA. This result could be explained by two possible reasons: first, vanoxerine occupied DA transporters which were the same binding sites of Taltirelin and thereby blocked the similar releasing effect or the entrance of Taltirelin into the cells (Baumann et al., 1994). Second, depletion of already limited DA pool by vanoxerine prevented the storage pool-dependent actions of Taltirelin, which is supported by the findings with reserpine. What’s more, we found that inhibition of DA synthesis using AMPT led to the reduction of Taltirelin-induced DA efflux by 84.08%. However, AMPT pre-treatment not only blocked the newly synthesis of DA, but also depleted the storage of DA (Butcher et al., 1988). Thus, the results supported the hypothesis that Taltirelin may have multiple action targets such as VMAT-2, DAT and TH, affecting DA efflux in multiple processes including the redistribution, uptake and synthesis of DA. Nevertheless, it is necessary to adopted more strategies such as genetic manipulation (Sulzer et al., 2005; Fleckenstein et al., 2007) to verified our hypothesis.

Our recent studies showed that Taltirelin raised TH levels in dyskinesia rats (unpublished), together with the results that AMPT blocked most DA-releasing action of Taltirelin, it is highly possible that Taltirelin could manipulate TH expression. Short-term (3 days) use of Taltirelin had no significant effect on TH level in the lesioned striatum of PD rats, whereas sub-chronic (7 days) injections significantly increased TH levels. In addition, incubation with Taltirelin (5 μM) for 24 h also greatly elevated the intracellular TH in primary striatal neurons, while TH remained unchanged in primary cortex neurons, indicating a neuron-type-dependent response or characteristic of Taltirelin actions. We further found that TH mainly co-localized with enkephalin in primary GABAergic neurons *in vitro*, whereas the dynorphin was almost undetectable. The sub-species of GABAergic neurons are directly related to the direct and indirect pathway model of PD (Calabresi et al., 2014), therefore, the influence of elevated TH on the basal ganglia neural circuits *in vivo* deserved further investigation. In addition, there are a small number of TH positive cells in the striatum under normal physiological conditions, which are in general identified as interneurons and do not contain AADC or DAT, i.e., non-dopaminergic neurons (Xenias et al., 2015). In our study, none of those TH-positive cells were stained with AADC or DAT either *in* or *ex vivo*, suggesting that Taltirelin did not induce the neuron type transformation at least in our experiment conditions. Regarding the functions of the TH-positive cells in the striatum, it has been proposed that those cells could co-synthesize DA with 5-HT neuronal terminals that contain ADDC (Keber et al., 2015; Kozina et al., 2017). Therefore, new-emerging TH-positive cells may help promoting DA synthesis and secretion in sub-chronic use of Taltirelin. ERK1/2 (or mitogen-activated protein kinase, MAPK) is a part of the Ras-Raf-MEK-ERK signaling pathway, participating in multiple important physiological activities (Jr, 2012). The activated form of ERK1/2, p-ERK1/2, is able to regulate the transcription and phosphorylation of TH (Tekin et al., 2014), and also could mediate the functions of TRH through TRH type 1 receptor (TRH-R1) (Choi et al., 2015), which is also the main action receptor of Taltirelin in the CNS (Thirunarayanan et al., 2013). In our study, p-ERK1/2 elevated after the treatment with Taltirelin, which could be blocked by MEK inhibitor U0126 (10 μM). However, p-ERK1/2 is a wide-functioning signaling factor and the expression of TH is subtly regulated by various factors, thus whether TRH or Taltirelin is able to increase phosphorylation level of TH or the affinity of TH cofactors (Lenartowski and Goc, 2011) still need further investigation.

**FIGURE 6:**
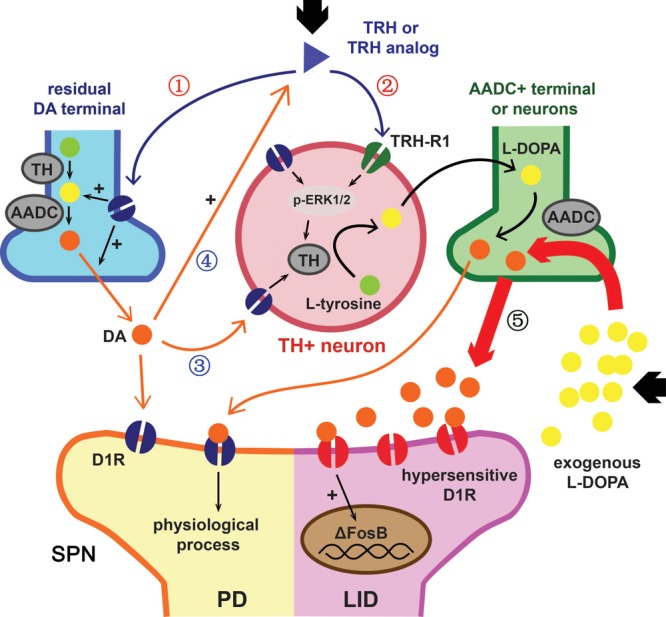
Hypothetical action mechanism of TRH or Taltirelin in PD model animals. Based on our results and recent literature, this scheme illustrated how TRH or its analog, Taltirelin (Tal) worked in PD model animals (adapted from Keber et al., 2015). Post administration, Tal firstly acted on the dopaminergic neurons on soma in the substantia nigra (SN) or on residual nerve terminal in striatum, resulting in increased dopamine (DA) synthesis and release (①). Secondly, after sub-chronic application, Tal bound to TRH type 1 receptor (TRH-R1) of striatal neurons, leads to an increase in tyrosine hydroxylase (TH) expression or activity mediated by p-ERK1/2. Striatal TH-positive neurons co-synthesized DA with 5-HT nerve terminals or other DOPA decarboxylase (AADC)-positive neurons and further increased DA levels (②). Elevated DA in turn stimulated TH expression in striatal neurons via dopamine type 1 receptor (D1R) or D2R (③) and further increased endogenous TRH release (④) (Przegaliñski et al., 1991). With such positive feedback, the level of DA in the striatum or cortex elevated slowly and steadily. On the contrary, exogenous L-DOPA usually caused a sudden increase of DA level in the striatum or cortex. Such pulse-like stimulus of DA caused hypersensitization of D1R and increased the expression of downstream ΔFosB, thus ultimately led to L-DOPA-induced dyskinesia (⑤).

ΔFosB, a truncated isoform of transcription factor FosB, is an established hallmark of dyskinesia. Due to its stable nature and tendency to accumulate, the level of ΔFosB in the striatum is positively correlated with the severity and frequency of dyskinesia. We once showed that overexpression of ΔFosB in the striatum of PD rats was enough to induce LID (Cao et al., 2010). L-DOPA caused a significant elevation of ΔFosB and showed an accumulating effect with injection times increased. Taltirelin group also showed similar cumulative effects, but the level was significantly lower than that of L-DOPA group. Studies once showed that L-DOPA significantly increased the level of TH and ΔFosB in the striatum of normal mice (Keber et al., 2015). In addition, striatal emerging TH-positive neurons usually appeared in areas of near complete or partial deprivation of DA rather than completely destructed areas (Lopez-Real et al., 2003). It has been suggested that such increase in TH-positive cells may be associated with D1R (Espadas et al., 2012), whereas DA exposure *in vitro* is known to increase the number of TH-positive cells in primary cortical and striatal cultures (Zhou et al., 1996). Thus, we wondered if that high-dose extraneous DA itself is enough to increase TH and ΔFosB in a dose-dependent effect and independent from LID. If this hypothesis is true, elevated ΔFosB in sub-chronic administration of Taltirelin without dyskinesia could be explained by the increased DA stimulated by Taltirelin, and the increased DA may also help promoting TH expression in striatum (Figure 6).

Whether the endogenic DA-releasing effect of Taltirelin would recede with the degeneration of dopaminergic terminals still needs further assessment in PD patients. However, in addition to the continuous DA-releasing effect that could avoid the occurrence of dyskinesia, Taltirelin also has other advantages over L-DOPA. First, a study recently reported a human-specific time-dependent pathological cascade of DA oxidation, mitochondrial and lysosomal dysfunction. The selective accumulation of neuromelanin (a complex aggregate consisted of oxidized DA, proteins, and lipids) in human neurons and the exogenous L-DOPA increased cytosolic DA, which may contribute to elevated mitochondrial oxidant stress, leading to a vicious cycle of dopamine and mitochondrial oxidation in human midbrain neurons (Burbulla et al., 2017). Consistently, we also observed a strikingly high level of DA in the intracellular contents of cortex and striatum of PD rats injected with L-DOPA. On the contrary, Taltirelin not only avoids the neurotoxicity of high-dose DA (Kang et al., 2018) but also provides disease-modifying effect which helps reserving residual dopaminergic neurons (Veronesi et al., 2007; Jaworska-Feil et al., 2010), which may help delaying the development of PD thus provides long-term benefit for the patients. Second, non-motor symptoms of PD, such as depression, constipation and cognitive dysfunction manifest usually earlier and can be equally or more disabling than the motor symptoms. Many non-motor symptoms do not respond to DA replacement therapy and some are even aggravated by this treatment (Poewe et al., 2017). Fortunately, TRH has been proved effect in improving depression (Szuba et al., 1996; Marangell et al., 1997; Zeng et al., 2007), memory impairment (Molchan et al., 1990) and regulating sympathetic nervous system (Khomane et al., 2011). These benefits associated with the role of TRH plays as homeostatic regulator in both CNS and periphery. For example, when the organism is sedated, TRH is a stimulant, but when the body is convulsed, TRH is anticonvulsant. Therefore, TRH and its analogs have great potential in treating many nervous system diseases (Khomane et al., 2011). However, it is impossible to fully explore the therapeutic potential, without thorough understanding of the mechanisms of action of TRH and relevant clinical trials to demonstrate its effectiveness. Above all, our study showed that TRH analog Taltirelin promoted DA release in the CNS in a mild and persistent manner, thus improving the motor function and relieving the PD-related abnormal electrical activities of PD rats without inducing dyskinesia in sub-chronic or high-dose use. Therefore, we introduced a novel promising drug for treating Parkinson’s disease.

## Author Contributions

CZ, GC, YX, ZZ, SP, KY, and XC conceived and designed the study. CZ, GC, YT, WZ, QP, JW, CC, XY, and SN performed the experiments. CZ and GC analyzed the data. CZ, GC, YX, SP, KY, and XC wrote the manuscript. All authors read and approved the final manuscript.

## Conflict of Interest Statement

The authors declare that the research was conducted in the absence of any commercial or financial relationships that could be construed as a potential conflict of interest.
