# Supplementary material for: TRH Analog, Taltirelin Improves Motor Function of Hemi-PD Rats Without Inducing Dyskinesia via Sustained Dopamine Stimulating Effect
Source: Front Cell Neurosci. 2018 Nov 13;12:417. doi: 10.3389/fncel.2018.00417 (PMC6282053; doi:10.3389/fncel.2018.00417)
Supplement: Supplementary file 1 [file Data_Sheet_1.docx]

**Supplementary materials**

**TRH analog, Taltirelin Improves Motor Function of Hemi-PD Rats Without Inducing Dyskinesia via Sustained Dopamine Stimulating Effect**

**Supplementary Figures**


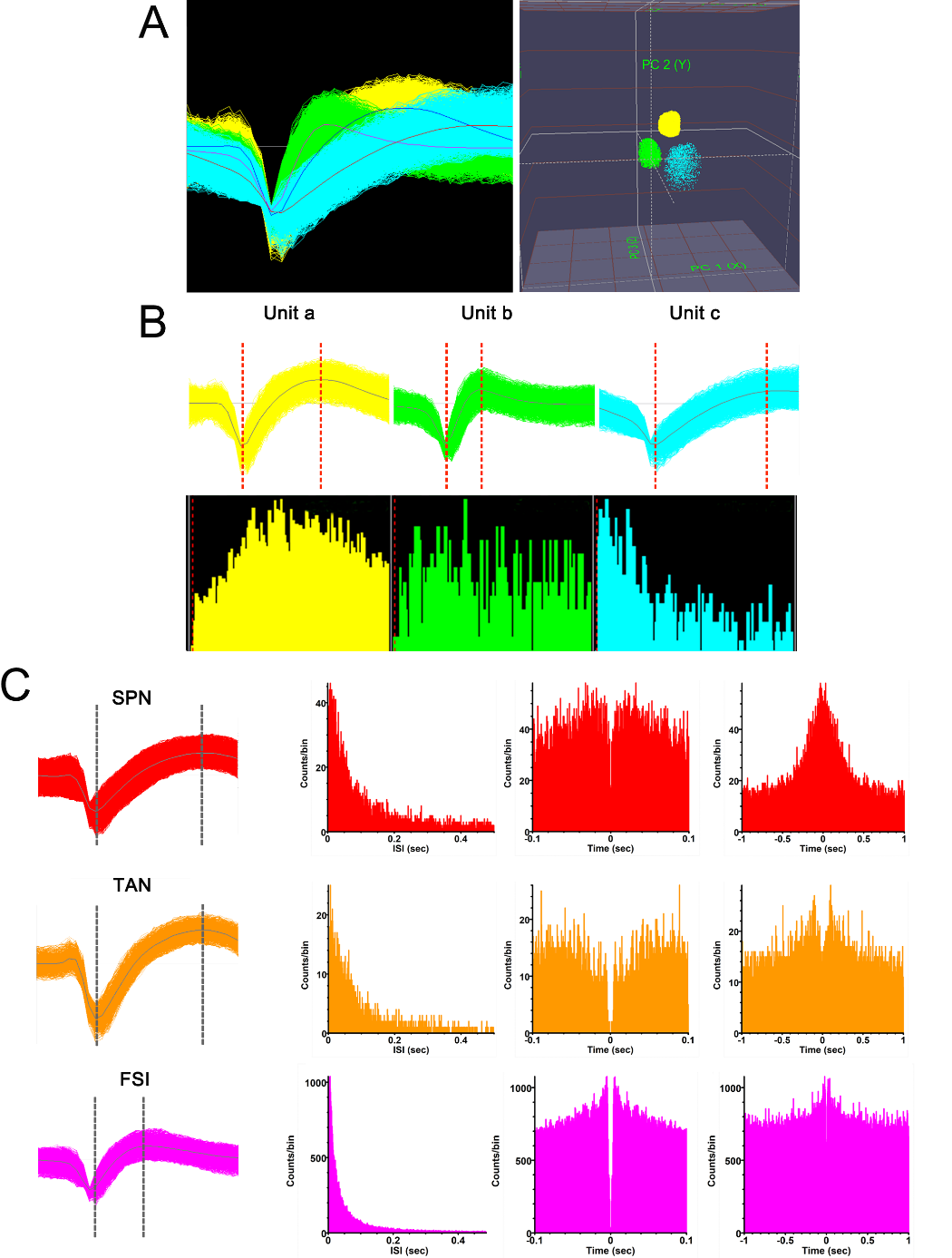


**Figure S1**. Single Unit Sorting and Classification. **(A)** Single-unit spike sorting procedure in Off-line Spike Sorter 2.8.5 workspace (Plexon Inc, Dallas, TX, USA). (left) Three units of waveform are recorded simultaneously in one channel in MI, (right) three units were isolated as distinct cluster in 3D space (PC1, PC2, and PC3); **(B)** (top) Typical single-unit spike templates representing Pyramidal projection neurons (PNs, Unit a) and interneurons (INs, Unit b), with dotted lines indicated spike width. (bottom) Corresponding ISI histogram; In general, PNs had much longer spike width and discharge at a lower rate (<10 Hz) compared with INs. **(C)** Spike recordings in dorsolateral striatum (DLS), from the left to right: typical single unit templates, ISI histogram, auto-correlagrams with ± 100 ms window/0.5 ms bin width and ± 1 s window/5 ms bin width. Striatal projection neurons (SPNs), tonically-active interneurons (TANs) and fast spiking interneurons (FSIs) were distinguished according to the following criteria: (i) FSI: short half-max width; high average firing rates. (ii) SPN: long half-max width; variable frequency; unimodal ISI histogram with typical high peak close to 0 s, and large variability of ISIs including some of 1 s or longer; autocorrelagrams is characterized by a central peak. (iii) TAN: half-max width usually longer than SPN; low average firing rates; autocorrelagrams is characterized by a central valley.


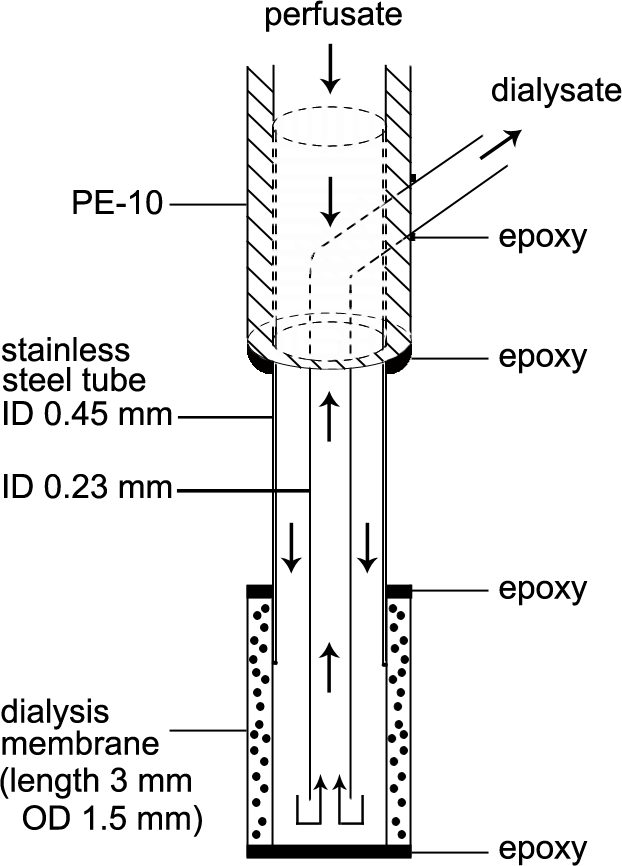


**Figure S2**. Self-made Concentric Circular Microdialysis Probe Schematic Diagram. Dialysis membrane, RC, cut-off molecular weight: 20 kDa, Union Carbide; membrane length: 3 mm, outside diameter 1.5 mm.


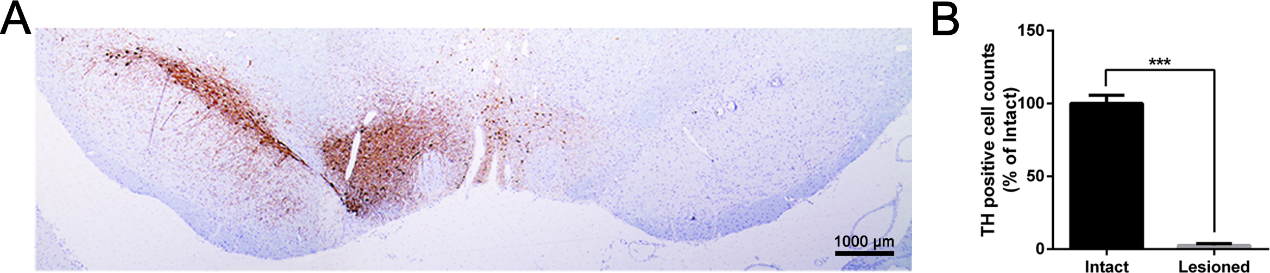


**Figure S3.** Tyrosine hydroxylase (TH) immunostaining in the substantia nigra (SN) of 6-OHDA-lesioned rat model. **(A)** Typical example of TH-immunostaining in the intact (left) and lesioned (right) SN of 6-OHDA-lesioned rat models. (B) Counts of TH-positive neurons. ****p* < 0.001. *N* = 3. Error bars represent SEM.

**
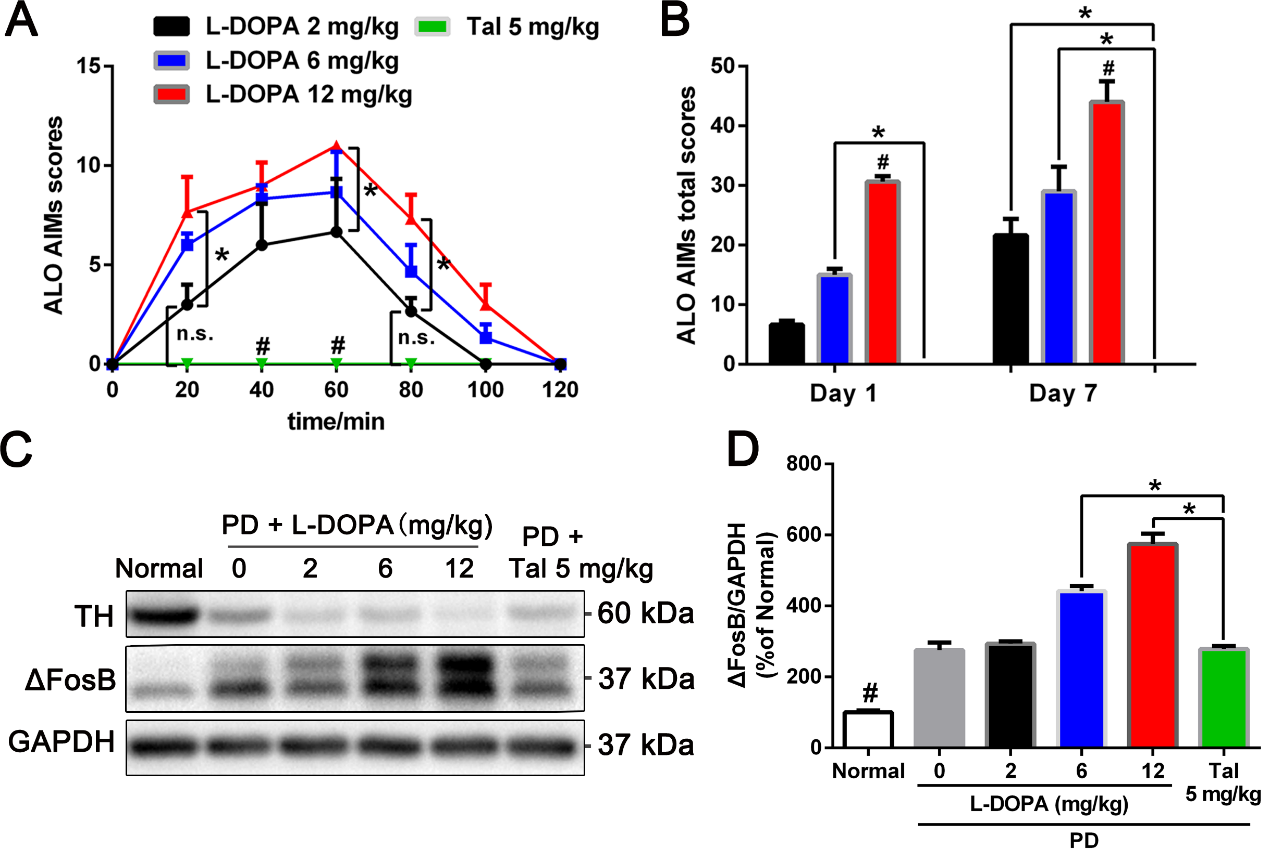
Figure S4.** Sub-chronic Administration of Taltirelin Induced No Behavioral or Biochemical Changes Related to Dyskinesia. The 6-OHDA-induced hemi-PD rats were given NS, L-DOPA 2, 6, 12 mg/kg (+ benserazide 6 mg/kg) or Tal 5 mg/kg i.p. injection once daily for 7 days. (**A**) ALO (axial, limb, and orofacial) AIMs scores were obtained every 20 min over 120 min following injection on the 7^th^ day. (**B**) ALO AIMs total scores were the sum of ALO AIMs scores of every 20 min during 120-min experiment on the 1^st^ and 7^th^ day. **(C-D)** Western blot analysis of tyrosine hydroxylase (TH) and ΔFosB in the DA-denervated striatum of each group. # *p* < 0.01 vs. other groups; * *p* < 0.05. *N* = 3; ns = no significance. Error bars represent SEM.





**Figure S5.** Measurement of serum thyroid hormones in 6-OHDA-induced hemi-PD rats. The 6-OHDA-induced hemi-PD rats were given NS, 1, 2.5 or 5 mg/kg of Taltirelin i.p. injection and blood was collected 2 h later. ELISA was used to measure the levels of different substances. **(A)** Serum thyroid stimulating hormone (TSH) concentrations; **(B)** Serum total triiodothyronine (TT3) concentrations; **(C)** Serum total thyroxine (TT4) concentrations; **(D)** Serum free T3 (FT3) concentrations; **(E)** Serum free T4 (FT4) concentrations. ***p* < 0.01 vs. control. *N* = 3. Error bars represent SEM.

**Supplementary video**

Video showed the behaviors of 6-OHDA-induced hemi-parkinsonian rats after the injection of Taltirelin (1 or 5 mg/kg). The behaviors of rats with Taltirelin (1 mg/kg) were virtually identical to the control, while the ones with higher dose (5 mg/kg) exhibited involuntary shaking and repeated jaws action.
